# Technical Challenges and Surgical Considerations in Sacrospinous Ligament Fixation for Apical Prolapse Repair

**DOI:** 10.3390/jcm15114209

**Published:** 2026-05-29

**Authors:** Stavros Athanasiou, Anastasia Prodromidou, Dimitrios Zacharakis, Aristotelis-Marios Koulakmanidis, Giuseppe Mascellino, Athanasios Douligeris, Nikolaos Kathopoulis, Themistoklis Grigoriadis

**Affiliations:** Urogynecology Unit, 1st Department of Obstetrics and Gynaecology, “Alexandra” General Hospital, National and Kapodistrian University of Athens, 11528 Athens, Greece; aprodromidou@med.uoa.gr (A.P.); dimzac@hotmail.com (D.Z.); aristoteliskoulak@gmail.com (A.-M.K.); mascellinog@gmail.com (G.M.); thanosdouligeris92@gmail.com (A.D.); nickatho@gmail.com (N.K.);

**Keywords:** sacrospinous ligament fixation, pelvic organ prolapse, vault prolapse, unilateral fixation, bilateral fixation, anterior approach, posterior approach, anchor-based devices, pelvic reconstructive surgery, native tissue repair

## Abstract

**Background/Objectives:** Sacrospinous ligament fixation (SSLF) is a well-established native tissue vaginal procedure for uterine/vault prolapse. Despite favorable success rates, the procedure presents technical challenges due to the deep operative field and proximity to critical neurovascular structures. To review current evidence regarding anatomical considerations, surgical technique, fixation strategies, suture materials, device-assisted methods, and perioperative complications in SSLF. **Methods:** A structured narrative review of the contemporary literature was conducted, focusing on comparative and systematic studies evaluating unilateral versus bilateral fixation, anterior versus posterior approach, suture type and number, and suture-capturing or anchor-based devices. Anatomical, functional, and safety outcomes were critically analyzed. **Results:** SSLF achieves favorable anatomical success rates with significant symptom improvement. Meticulous knowledge of sacrospinous ligament anatomy is critical to reduce bleeding and neuropathic complications. Unilateral fixation remains the most common technique, while bilateral fixation may benefit selected patients. According to the available evidence, the anterior approach may better preserve vaginal length, although it may be associated with longer operative time and short-term urinary morbidity. Absorbable and permanent sutures appear to provide comparable anatomical durability, while placement of two sutures remains the most commonly used fixation strategy. Device-assisted techniques may facilitate suture placement but require advanced anatomical expertise. **Conclusions:** SSLF is a safe and effective suspension procedure when individualized and meticulously performed. Further randomized studies evaluating long-term anatomical and patient-reported outcomes are warranted.

## 1. Introduction

Apical prolapse refers to the descent of the vaginal apex involving either the cervix or the vaginal cuff following hysterectomy [1]. According to the Pelvic Organ Prolapse Quantification (POP-Q) system endorsed by the International Continence Society (ICS), apical prolapse is assessed based on the position of point C (cervix or vaginal cuff) relative to the hymen and total vaginal length as determined by the most distal point [2]. Apical prolapse is a common condition, affecting approximately 50% of parous women on clinical examination, while it is symptomatic in approximately 10–20% [3]. The vaginal apex plays a central role in supporting the anterior and posterior compartments. Consequently, restoring apical support is essential in achieving durable prolapse repair [1].

Vaginal surgery represents an effective approach for the management of apical prolapse, aiming to restore anatomy. Various transvaginal surgical techniques have been described, among which sacrospinous ligament fixation (SSLF) was first described by Sederl in 1958 [4]. Since its introduction, SSLF has become a widely utilized native tissue vaginal procedure for the management of apical prolapse [5]. Alongside uterosacral ligament suspension and abdominal or minimally invasive sacrocolpopey, SSLF represents one of the principal surgical options for restoration of apical support. It offers several advantages, including avoidance of peritoneal entry, thereby reducing the risk of intra-abdominal complications [6], as well as shorter operative time, reduced blood loss, and shorter hospital stay [7]. Reported anatomical outcomes show apical cure rates of approximately 80–90% in various studies [8,9]. In addition, SSLF has been associated with significant improvements in prolapse-related symptoms, quality of life and sexual function [10]. However, SSLF presents specific surgical challenges mainly based on the fact that the procedure is performed in a deep and confined operative field, requiring careful dissections and familiarity with complex pelvic anatomy. The sacrospinous ligament lies in close proximity to critical neurovascular structures and the rectum, increasing the risk of injuries during surgery [5,6]. Furthermore, variations in the described SSLF techniques introduce additional considerations, as technical precision and surgical judgment may significantly influence both procedural safety and postoperative outcomes.

Despite the widespread use of SSLF, important technical variations remain regarding surgical approach, fixation strategy, suture material, and use of device-assisted systems. In addition, the available literature remains heterogeneous, while several aspects of technique selection, complication prevention, and functional outcomes continue to be debated. This narrative review aims to provide an updated overview of the technical challenges and surgical considerations of SSLF that could be clinically relevant for surgeons involved in pelvic reconstructive surgery.

## 2. Methods

A structured narrative review of the literature was conducted using PubMed/MEDLINE, Scopus, and Google Scholar databases to identify relevant studies evaluating sacrospinous ligament fixation (SSLF) for apical prolapse repair. Our search terms included combinations of the following keywords: “sacrospinous ligament fixation,” “apical prolapse,” “vaginal vault prolapse,” “anterior approach,” “posterior approach,” “bilateral fixation,” “suture material,” “anchor-based devices,” and “suture-capturing devices.” Studies written in the English language published up to March 2026 were considered. Randomized controlled trials, comparative observational studies, systematic reviews, cadaveric studies, and large case series relevant to surgical technique, anatomical considerations, functional outcomes, and perioperative complications were included. Priority was given to contemporary studies and publications with direct relevance to technical aspects of SSLF.

## 3. Relevant Anatomy and Anatomical Considerations in SSLF

A precise understanding and knowledge of the anatomical structures in the deep pelvis is considered of critical importance for the performance of SSLF. The sacrospinous ligament (SSL) is a dense connective tissue band that extends from the ischial spine laterally to the lower margins of the sacrum and coccyx medially [6]. It is located posterior to the ischial spine, where it is covered by the coccygeus muscle, forming the coccygeus muscle–sacrospinous ligament (C-SSL) complex, which may be recognized by pelvic examination [7]. This represents the key anchor point for vaginal apical suspension procedures. The length of the complex varies (30–65 mm), with an average thickness of approximately 3 mm.

The nerves supplying the coccygeus and levator ani muscles lie in close proximity to the SSL [8]. In particular, the S3 and S4 nerve roots typically run over the superior and medial borders of the C-SSL, while the pudendal nerve courses lateral to the ligament and may lie within 2.5 cm of the ischial spine [8]. Based on cadaveric studies, it is recommended that sutures should be placed 15–30 mm medial to the ischial spine to minimize the risk of neurovascular injury [8,9]. Inadequate exposure or imprecise suture placement may lead to inadvertent nerve involvement, which can manifest clinically as postoperative gluteal or perineal pain, sensory or motor dysfunction, or bowel symptoms.

Vascular anatomy constitutes an equally critical consideration during SSLF. The internal pudendal artery (IPA), a branch of the anterior division of the internal iliac artery, courses posterior to the ischial spine; cadaveric studies demonstrate that, in nearly 90% of specimens, it lies within 1.5 mm of the spine [9]. The inferior gluteal artery (IGA) may arise from the anterior or posterior division of the internal iliac artery or share a common trunk with the IPA. It traverses the posterior surface of the mid-portion of the coccygeus–sacrospinous ligament complex and is frequently located only 3–5 mm superior to the ligament [10]. Equally important is the venous anatomy. The internal pudendal and inferior gluteal veins accompany their corresponding arteries and drain into the internal iliac vein, forming a dense venous plexus posterior to the ischial spine [7]. Owing to their thin walls, these veins are particularly susceptible to traction, needle penetration, or blind suture placement. Venous injury may lead to brisk but non-pulsatile bleeding or to delayed retroperitoneal and pararectal hematoma formation. Given this close arterial and venous proximity, SSLF carries a small but significant risk of major hemorrhage, with injury to the inferior gluteal vessels representing the most frequently reported vascular complication.

## 4. Surgical Technique and Variants

Sacrospinous ligament fixation involves the unilateral or bilateral suspension of the vaginal apex to the sacrospinous ligament–coccygeus muscle complex, with or without the use of grafts. The transvaginal, extraperitoneal approach represents the most commonly employed technique for SSLF. This approach offers the additional benefit of avoiding entry into the peritoneal cavity, which is particularly advantageous in women with prior abdominal surgery or an increased likelihood of pelvic adhesions [11].

The sacrospinous ligament may be accessed through posterior, anterior, or apical vaginal incisions, with the posterior approach being most widely utilized. The ischial spine represents the primary anatomical landmark for surgical orientation. The sacrospinous ligament is then identified through either direct visualization and/or blunt digital dissection after mobilization of the overlying connective tissue to optimize identification and ensure adequate exposure of the ligament complex [12]. Concerning the posterior approach, following tissue hydrodissection, a vertical midline incision is made in the posterior vaginal wall. The rectovaginal fascia is dissected laterally to enter the pararectal space, with dissection continuing until the ischial spine is palpated [12,13]. This approach offers the advantage of a relatively straightforward dissection and allows for concomitant repair of posterior compartment defects. In the anterior approach, dissection of the vesicovaginal and paravaginal spaces allows access to the sacrospinous ligament through the mobilization of the bladder and lateral dissection along the arcus tendineus fascia; this approach also facilitates concomitant anterior compartment repair, potentially reducing the risk of cystocele recurrence [14]. Anterior SSLF appears to better preserve total vaginal length and maintain a more horizontal vaginal axis, which may reduce anterior recurrence and postoperative dyspareunia associated with the posterior approach [15]. Nonetheless, the anterior dissection can be technically challenging and may increase the risk of urinary dysfunction, particularly when performed bilaterally.

Following adequate exposure of the ligament, one or two permanent or delayed-absorbable sutures are placed through the ligament 1.5–2.5 cm medial to the ischial spine, taking care to avoid full-thickness bites that may incorporate adjacent neurovascular structures. When direct visualization is achieved, suture placement may be facilitated using instruments such as the Deschamps ligature carrier or Miya hook [15]. To minimize the extent of dissection required for direct exposure, several suture-capturing devices have been developed, including the i-Stitch device, Endo Stitch^TM^ (Medtronic, Minneapolis, MN, USA), and Capio SLIM Suture Capturing Device systems (Boston Scientific Corporation, Marlborough, MA, USA), which enable palpation-guided suture placement with less tissue mobilization [12]. In selected cases, sacrospinous ligament fixation has also been complemented with prosthetic or biologic graft in an attempt to improve anatomical durability, particularly in patients with advanced or recurrent prolapse [16,17]. Some studies have reported favorable anatomical outcomes with mesh-augmented SSLF techniques; however, interpretation of these findings remains limited by heterogeneous study design, variable mesh materials, and evolving regulatory concerns regarding transvaginal prosthetic use [16,18]. Furthermore, mesh-related complications, including exposure, pain, and dyspareunia, must be carefully balanced against potential anatomical benefits. Consequently, the role of prosthetic augmentation in SSLF remains individualized and should be considered cautiously within the context of current guidelines, patient characteristics, and surgeon expertise.

The suspension sutures are then secured to the fibromuscular layer of the vaginal apex, with or without incorporating vaginal epithelium. Particular attention is paid to maintain tension-free approximation in order to restore apical support without excessive shortening or axis distortion [12,15]. Concomitant anterior or posterior vaginal wall repair may be performed as indicated. A digital rectal examination is routinely performed prior to knot tying to exclude inadvertent rectal injury. Meticulous hemostasis is critical during the procedure, given the proximity of major pelvic vessels and the potential for significant hemorrhage.

### 4.1. Unilateral Versus Bilateral Sacrospinous Ligament Fixation

Unilateral fixation is the most common approach and is typically performed on the right side. The technique is associated with reduced operative time and technical complexity, as well as a lower theoretical risk of rectal injury owing to the anatomical position of the rectosigmoid colon on the left side. However, unilateral fixation creates an inherent biomechanical alteration by anchoring the vaginal apex to a single lateral point, resulting in deviation of the vaginal axis. Some authors have raised that unilateral fixation may be associated with an increased risk of anterior compartment prolapse [19], along with vaginal narrowing, dyspareunia, and bowel complaints [15]. Cespedes et al. reported that supporting the apex on both sides helps maintain a more natural, horizontal vaginal axis, improving the vagina’s ability to resist intra-abdominal pressure and potentially lowering the risk of recurrent prolapse and postoperative dyspareunia [15].

Only two studies have compared perioperative outcomes between unilateral and bilateral SSLF [20,21], as shown in Table 1.

In the randomized trial by Salman et al., no differences were observed in terms of operative time, blood loss, and hospital stay [20]. Both techniques resulted in significant anatomical improvement at 6 months, with low recurrence rates and no serious intra- or postoperative complications [20]. However, the trial was limited by the short follow-up, small sample size, and use of one type of fixation device (Anchorsure^®^, Neomedic International, Barcelona, Spain) [20]. In contrast, a retrospective comparative study by Jones et al. found that the unilateral group had significantly longer operative times (127.4 vs. 105.7 min, *p* = 0.01) and greater blood loss (270.7 vs. 200.8 mL, *p* = 0.02) compared to the bilateral fixation group [21]. However, bilateral fixation procedures were performed after the unilateral cases, suggesting that the improved perioperative outcomes may in part reflect progression along the surgical learning curve. Length of hospital stay and anatomic success were not different [21]. Cure rates exceeded 85% in both groups, and complications were rare [21]. Overall, anatomical outcomes and safety appear broadly comparable between unilateral and bilateral SSLF, although perioperative efficiency may depend on surgical technique and device use.

Limited observational data suggest that bilateral SSLF may be associated with high anatomical success and low recurrence rates, even in patients with advanced prolapse. Published series report anatomical success rates up to 80% at 2 years, with low rates of prolapse recurrence, supporting bilateral SSLF as a durable option in complex cases of advanced prolapse and multicompartment involvement with an inherently higher risk of failure [22,23,24]. Despite the encouraging outcomes in terms of anatomical success, bilateral SSLF remains less frequently performed than unilateral suspension. The reasons are partly related to the fact that the bilateral technique is more technically challenging, could take longer, and carries a theoretical risk of a higher risk of nerve, vessel injury and rectal injury [22,23,24].

However, interpretation of these findings should remain cautious, as the available evidence is limited by small sample sizes, heterogeneous surgical techniques, variable use of fixation devices, and relatively short follow-up periods. Furthermore, most comparative data derive from retrospective or non-randomized studies, limiting the ability to establish a clear superiority of one approach over the other. Further, larger randomized trials with longer follow-up and standardized techniques are warranted to clarify the potential clinical advantage of one approach over the other.

### 4.2. Anterior Versus Posterior Approach

The sacrospinous ligament for SSLF may be accessed through dissection of either the anterior or the posterior vaginal wall. Three comparative studies have evaluated the perioperative outcomes after the anterior and posterior approaches for SSLF (Table 2).

In the early series by Goldberg et al., the anterior approach was associated with a significantly greater vaginal length (*p* = 0.01), with a mean difference of 0.76cm in favor of anterior SSLF [25]. Additionally, reoperation rates were significantly lower in the anterior group (3.9% vs. 15.2%, *p* = 0.01), whereas rates of postoperative lower abdominal pain, back pain and dyspareunia were comparable between groups [25]. Similarly, according to the prospective comparative study by Bastani et al., patients in the anterior group had a significantly greater total vaginal length postoperatively (*p* < 0.001) compared with those undergoing posterior SSLF. One case of recurrence (1.5%) was observed in the posterior group [26]. No major intraoperative or postoperative complications were reported, while patients’ satisfaction among the two groups was comparable [26]. In the most recent comparative study by Şahin et al., despite the fact that no significant difference was observed in absolute postoperative point C measurements between groups, the change from pre- to postoperative point C was significantly greater in patients who underwent anterior SSLF compared to those for posterior SSLF [27]. The anterior approach was associated with significantly longer operative time (80 vs. 42.5 min, *p* < 0.001); however, this was not reflected in length of hospital stay, estimated blood loss and recurrence rates [27]. Low complication rates were recorded for both groups. More recently, Mezes et al. presented a comparative cohort evaluating anterior versus posterior sacrospinous hysteropexy. The anterior approach was associated with a significantly longer time to recurrence of patient-reported bulge symptoms compared with the posterior approach [28]. On multivariable analysis, a posterior approach was independently associated with a higher risk of earlier bulge symptom recurrence (hazard ratio 5.85) [28]. Notably, the anterior approach was associated with a higher rate of short-term, predominantly low-grade postoperative complications, mainly urinary retention and urinary tract infections, likely reflecting the high rate of concomitant anterior compartment repair. Despite the reported superiority of the anterior approach in anatomical parameters, particularly postoperative vaginal length, there is currently no evidence to support a corresponding improvement in functional outcomes or clinically meaningful benefits.

Selection of the surgical approach should therefore balance technical complexity, compartmental outcomes, and potential functional sequelae. Significant heterogeneity exists regarding patient selection, concomitant procedures, surgical technique, and outcome reporting among the available studies, making direct comparison difficult. Consequently, the reported anatomical advantages of the anterior approach should be interpreted cautiously until validated by larger prospective randomized studies with long-term functional assessment to clarify the true clinical benefits of anterior versus posterior fixation, with longer follow-up.

## 5. Suture Material and Fixation Strategy

### 5.1. Type of Suture: Absorbable Versus Permanent

With respect to suture, the use of permanent monofilament (e.g., polypropylene) or multifilament (e.g., polyester/silk) sutures may provide a durable apical support. However, this results in the permanent presence of foreign material at the apex, which may increase the risk of suture exposure, granulation tissue formation, or chronic pain. On the other hand, delayed-absorbable monofilament (e.g., PDS) or multifilament (e.g., Vicryl) sutures aim to minimize foreign-body sequences, while long-term support relies on tissue remodeling. According to a retrospective study by Padoa et al., the choice between absorbable and permanent sutures does not significantly influence the success of SSLF [29]. More specifically, the 12-month anatomic success was comparable between the two groups (69% absorbable vs. 67% permanent) with similar subjective cure [29]. However, immediate postoperative pain was significantly higher in the permanent sutures group (postoperative day 1 according to VAS, *p* = 0.01). This difference resolved by 12 months in all patients. In addition, the absorbable suture group exhibited lower estimated blood loss (*p* = 0.016) [29]. Similarly, a large 2023 systematic review and meta-analysis of 11 studies and 792 patients, evaluating the use of permanent versus absorbable sutures for apical prolapse surgeries (including SSLF), found no difference in anatomic success according to suture type [30]. Permanent sutures, however, were associated with a higher incidence of suture-related complications, including increased risks of suture exposure, need for reoperation due to suture complications, and granulation tissue formation. Most studies (73%) used permanent sutures, but the majority of patients (77%) actually underwent fixation with absorbable sutures (609 vs. 183). This reflects the heterogeneity in study design and surgeon preference, as well as a shift toward the use of absorbable material in clinical practice.

### 5.2. Number of Sutures

Concerning the number of sutures, the most common practice is the placement of two sutures at the ligament attachment site. According to a recent systematic review, 72.7% of SSLF studies used two sutures, most often unilateral (right-sided) [30]. Currently, the available literature comparing single- versus double-suture techniques in SSLF is limited. There is a registered retrospective cohort (NCT07106866) study aiming to compare the traditional double suture SSLF technique with a modified loop suture method [31]. The results of this trial are anticipated to clarify whether the loop suture technique offers anatomical durability comparable to the conventional double-suture approach, with a potential reduction in morbidity.

### 5.3. Suture-Capturing and Anchor-Based Devices

To address the technical challenges of deep pelvic suture placement, novel tools such as suture-capturing devices and anchor-based systems have been increasingly adopted. These tools aim to optimize exposure, minimize dissection, and potentially facilitate the adoption of bilateral SSLF without compromising safety or effectiveness [32]. Most suture-capturing and anchoring devices are designed to enable suture placement without the need for full direct visualization of the sacrospinous ligament [10,33]. Although this may reduce operative time and tissue trauma, it may also result in less precise anatomic placement, thus increasing the risk of nerve entrapment or vascular injury.

The Capio SLIM Device—a compact, trocar-free instrument—facilitates precise “push-and-catch” suture placement with minimal dissection [34]. In cadaveric studies, data suggest that the Capio device may have an acceptable safety profile with placement without vascular penetration [33,34]. More recently, anchor-based systems such as Anchorsure^®^ and the Saffron Fixation System have been introduced. The study by Evangelopoulos et al. compared the anchor-based devices with suture-capture ones and found similar perioperative complication rates with only modest differences in early postoperative anatomical outcomes favoring anchor-based devices [35]. This was also confirmed by the prospective study by McKenzie et al., indicating that both anchor-based and suture capture devices offered similar patient-reported improvements and anatomical durability, with low pain and failure rates [36].

In contrast, Amiri et al. conducted a systematic review and meta-analysis of 125 studies encompassing 10,216 cases of SSLF performed with the use of any of the suture-capturing or anchor-based devices [32]. Overall reoperation rates were low (1.2%), but notably higher in procedures utilizing the Shutt Suture Punch System (ConMed Linvatec, Utica, NY, USA), Laurus (Laurus Labs, Hyderabad, India), and Anchorsure^®^ devices. The Capio device was associated with the highest incidence of nerve injury, while the Laurus or Raz anchoring systems were associated with increased hematoma rates. Interestingly, the authors concluded that suture-capturing devices are associated with higher complication rates compared to traditional suture techniques. This finding highlights the necessity for comprehensive knowledge of pelvic anatomy, appropriate surgical experience, and the ability to promptly identify and address device-related complications.

Overall, although suture-capturing and anchor-based devices may facilitate suture placement and reduce the extent of surgical dissection, reduced dissection should not necessarily be interpreted as improved procedural safety. Available evidence regarding these devices remains heterogeneous and is largely based on retrospective and observational studies. Importantly, based on the increased complication rates with some devices, device-assisted techniques still require precise anatomical knowledge and appropriate surgical training, as inaccurate placement may increase the risk of neurovascular injury, hematoma formation, or nerve entrapment. Therefore, device selection should be individualized according to surgeon experience, familiarity with the specific system, and patient anatomical characteristics, rather than based solely on perceived technical simplicity.

## 6. Intraoperative and Postoperative Challenges

Despite the fact that SSLF is considered a safe and effective procedure for the management of apical prolapse, it carries perioperative risks that deserve careful consideration. When performed by experienced individuals, complications are rare, yet their recognition and management are critical for optimizing outcomes and counseling patients.

### 6.1. Bleeding Risk

Intraoperative bleeding represents the main concern during SSLF, given the close proximity of the SSLF to the inferior gluteal, pudendal, and middle rectal vessels [6,37]. Bleeding-related complications are the most common indication for reoperation and hospital readmission following SSLF [37]. Injury to the hypogastric and pudendal venous plexus may cause significant hemorrhage, which can be challenging to control in the deep pelvis under conditions of limited visualization [38]. The reported transfusion rates in large series range from 0 to 2% [13,38]. Additionally, surgeons should be cautious of the potential anatomical variations, as major vessel injury, although rare, has been described in case reports [33].

### 6.2. Nerve Injury and Postoperative Buttock Pain

Buttock or posterior thigh pain is a common complaint during the early postoperative period following SSLF, reported by up to 84% of women [39,40]. While symptoms generally resolve by 6 weeks, persistence may suggest pudendal nerve entrapment related to the applied sutures, with an estimated incidence of 1–2% [40]. This complication occurs when a suture inadvertently compresses the pudendal nerve or its branches, resulting in debilitating pain, numbness, or paresthesia in the gluteal, perineal, or genital regions. When postoperative focal buttock or perineal pain raises suspicion of neural involvement, prompt evaluation—including consideration of suture removal—may effectively relieve symptoms without necessarily compromising apical support [39]. In addition to this, early recognition and management of suspected neuropathic pain are important in order to reduce the risk of persistent sensitization and chronic pain development. Initial conservative management may include neuropathic pain medication, such as gabapentinoids or antidepressants, while further evaluation is undertaken and the need for surgical suture removal is assessed. In a retrospective analysis of 21 women who presented with severe, persistent neuropathic pain following SSLF, suture removal was shown to be both safe and effective [39]. Suture removal was performed after a median interval of 414 days postoperatively (range 8–1855 days) [39]. Following removal, up to 95% of patients experienced pain relief, while 57% reported complete pain resolution. Complete suture removal was achieved in 86% of cases [39]. One case of notable intraoperative bleeding (520 mL) occurred during suture removal, but no transfusion was required [39]. At 6–8 weeks follow-up, only two patients developed recurrent anatomically significant prolapse (POP stage ≥ 2), warranting repeat prolapse surgery [39].

### 6.3. Urinary and Defecatory Dysfunction

In the long-term, urinary dysfunction following SSLF may present as de novo urge urinary or stress urinary incontinence (UUI or SUI). While postoperative incontinence could be attributed to the unmasking of pre-existing occult stress incontinence, it may also be related to changes in urethrovesical dynamics or nerve irritation, particularly in anterior approaches that require dissection into the paravesical space [18]. According to the analysis by Gagyor et al., while SSLF significantly alters anatomical support by reducing urethrovesical junction mobility, these changes do not appear to influence the onset or progression of SUI within the first two postoperative years, suggesting that other factors may play a more important role in postoperative SUI development [41]. Reported rates of de novo SUI vary across studies but generally range between 5% and 10% [42,43].

Defecatory dysfunction, including incomplete evacuation, obstructed defecation, or new-onset constipation, has also been described, particularly when fixation alters the vaginal axis or compresses the rectal nerve supply. Despite the fact that a small proportion of patients develop de novo fecal incontinence after SSLF, other studies suggest that the procedure may improve pre-existing fecal incontinence [13,44]. It remains challenging to directly attribute the improvement of anal incontinence to SSLF, given the absence of controlled studies. A possible explanation lies in the integral theory, which suggests that connective tissue defects of the anterior and posterior suspensory ligaments may contribute to idiopathic fecal incontinence [44]. Individualization of surgical repair based on each patient’s needs is of critical importance for selecting the optimal approach according to compartmental symptoms and preoperative functional assessment.

### 6.4. Suture Erosion, Dyspareunia, and Sexual Function

Permanent sutures have been associated with a higher risk of exposure and granulation tissue formation, with a recent meta-analysis reporting approximately a 6% higher incidence of exposure when permanent sutures are used compared with absorbable ones [30]. Patients may present with spotting, discharge, or focal tenderness. Management is usually conservative and includes office-based trimming or, when necessary, surgical excision of the exposed suture material.

De novo dyspareunia following SSLF remains a matter of debate. Some studies report new-onset painful intercourse in 3.3 to 7% of patients, while others found no significant difference in sexual function compared to the preoperative status [44,45]. In contrast, a prospective study of 26 sexually active women undergoing bilateral sacrospinous ligament fixation with vaginal hysterectomy reported a significant postoperative reduction in dyspareunia [46]. In this study, sexual function improved across all Pelvic Organ Prolapse/Urinary Incontinence Sexual Questionnaire (PISQ-12) domains, which was attributed to symptomatic relief from prolapse, correction of vaginal axis deviation, and the restoration of normal anatomy [46]. Overall, women reported improved sexual life after surgery, largely due to reduced discomfort and avoidance behavior associated with prolapse. Consequently, careful attention to suture tension and preservation of an appropriate vaginal axis alignment are essential for optimizing postoperative sexual outcomes.

## 7. Conclusions and Future Directions

Sacrospinous ligament fixation remains a well-established and effective native tissue vaginal procedure for the management of apical pelvic organ prolapse. When appropriately selected and meticulously performed, SSLF provides durable anatomical support with acceptable morbidity and meaningful symptomatic improvement.

Successful outcomes depend largely on detailed knowledge of deep pelvic anatomy, careful suture placement, appropriate tensioning, and avoidance of neurovascular injury. Selection of unilateral versus bilateral fixation, anterior versus posterior approach, and type of suture or device type should be individualized according to patient anatomy, prolapse severity, functional symptoms, prior surgical history, and surgeon experience.

Although device-assisted techniques may facilitate suture placement, their safe use still requires substantial anatomical expertise and familiarity with the specific system employed. Current evidence comparing different SSLF techniques remains limited by heterogeneous study design, retrospective data, and relatively short follow-up periods.

For this reason, structured training, mentorship, and gradual progression in case complexity are fundamental to achieving and maintaining proficiency in SSLF. Simulation-based education and cadaveric dissection courses may further enhance anatomical understanding and technical confidence, particularly for surgeons adopting newer devices or bilateral techniques.

Future well-designed prospective and randomized comparative studies with long-term anatomical and patient-reported outcomes are needed to better define the optimal surgical approach, fixation strategy, and role of emerging device-assisted techniques in SSLF. In addition, future research may further explore the evolving role of sacrospinous ligament fixation techniques in uterine-preserving prolapse surgery. Sacrospinous hysteropexy has emerged as an increasingly utilized option for selected women seeking uterine preservation, although further comparative studies evaluating long-term anatomical, functional, and patient-reported outcomes are still needed.

## Figures and Tables

**Table 1:** Study, patient characteristics and main outcomes (BSSLF vs. USSLF).

Year; Author	Design	N	Technique	OT (min)	EBL (mL)	LOS (Days)	Anatomical Outcomes	Complications
2019; Salman et al. [20]	PS-RCT	93 (52 vs. 41)	VH + SSLF Anchorsure^®^	76.6 ± 10.7 vs. 80.5 Time to fixation (NS)	133 ± 40.9 vs. 140 ± 50.8 (NS)	2.3 ± 0.9 vs. 2.4 ± 0.8 (NS)	Vault recurrence 2 cases (3.8%) vs. 0; cystocele 1 vs. 3 (asymptomatic)	No
2001; Jones et al. [21]	RS	103 (41 vs. 62)	Posterior SSLF with typical concomitant repairs	127.4 ± 50.3 vs. 105.7 ± 36.8 (*p* = 0.01)	270.7 ± 166.2 vs. 200.8 ± 140.6 (*p* = 0.02)	2.8 ± 1.2 vs. 2.8 ± 3.2 (NS)	Cure: 90.2% vs. 85.5% (*p* = 0.56)	No reoperations: 2 vs. 1

USSLF: Unilateral Sacrospinous Ligament Fixation; BSSLF: Bilateral Sacrospinous Ligament Fixation; RCT: Randomized controlled trial; VH: Vaginal hysterectomy; OT: Operative time; EBL: Estimated blood loss; LOS: Length of stay; NS: Not significant.

**Table 2:** Study, patient characteristics and main outcomes (anterior vs. posterior SSLF).

Year; Author	Design	N	Technique	Perioperative Outcomes	Anatomical Outcomes	Complications	Reoperation
2001; Goldberg et al. [25]	RS	168 (76 vs. 92)	Two permanent sutures (00)	N/A	TVL: 9.08 vs. 8.33 cm, *p* = 0.002 Point C: −8.51 vs. −6.60cm, *p* = 0.04	N/A	3.9% vs. 15.2%, *p* = 0.01
2022; Bastani et al. [26]	PS	135 (68 vs. 67)	Vicryl-0 Capio device Bilateral vs. Unilateral	Hospital stay1 ± 0.2 vs. 2.8 ± 1.6 *p* < 0.0001	TVL: 12.2 ± 1.2 vs. 7.8 ± 0.6 cm, *p* < 0.001 Patient satisfaction: 94.1% vs. 95.5%, *p* > 0.05	0 vs. 0	0 vs. 1
2023; Şahin et al. [27]	RS	48 (24 vs. 24)	Bilateral vault prolapse (anchor system)	OT: 80 vs. 42.5, *p* < 0.001 LOS: 2 vs. 1, *p* = 0.105 EBL: 80 vs. 80, *p* = 0.059	C: 7.67 ± 3.51 vs. 5.92 ± 3.14, *p* = 0.028	Urinary dysfunction in anterior approach; *p* = 0.012	Recurrence 16.7% vs. 16.7%

SSLF: Sacrospinous Ligament Fixation, OT: Operative time (min), LOS: Length of stay (days), EBL: Estimated blood loss (mL), RS: Retrospective, PS: Prospective, TVL: Total vaginal length, N/A: not available.

## Data Availability

No new data were created.

## Abbreviations

The following abbreviations are used in this manuscript:

ICS	International Continence Society
SSLF	Sacrospinous ligament fixation
C-SSL	Coccygeus muscle–sacrospinous ligament
PISQ-12	Pelvic Organ Prolapse/Urinary Incontinence Sexual Questionnaire
UUI	Urge urinary incontinence
IPA	Internal pudendal artery
IGA	Inferior gluteal Artery
SUI	Stress urinary incontinence
RCT	Randomized controlled trial
RS	Retrospective
PS	Prospective
TVL	Total vaginal length
EBL	Estimated blood loss
LOS	Length of stay
UTI	Urinary tract infections
POP-Q	Pelvic organ prolapse quantification
VAS	Visual analog scale
POP	Pelvic organ prolapse

## References

[1] Brubaker L., Maher C., Jacquetin B., Rajamaheswari N., von Theobald P., Norton P. (2010). Surgery for pelvic organ prolapse. Urogynecology.

[2] Abrams P., Cardozo L., Fall M., Griffiths D., Rosier P., Ulmsten U., van Kerrebroeck P., Victor A., Wein A.J. (2003). The standardisation of terminology in lower urinary tract function: Report from the standardisation sub-committee of the International Continence Society. Urology.

[3] Nygaard I., Barber M.D., Burgio K.L., Kenton K., Meikle S., Schaffer J., Spino C., Whitehead W.E., Wu J., Brody D.J. (2008). Prevalence of symptomatic pelvic floor disorders in US women. JAMA.

[4] Sederl J. (1958). Zur Operation des Prolapses der blind endigenden Scheide. Geburtshilfe Frauenheilkd..

[5] Florian-Rodriguez M.E., Hare A., Chin K., Phelan J.N., Ripperda C.M., Corton M.M. (2016). Inferior gluteal and other nerves associated with sacrospinous ligament: A cadaver study. Am. J. Obstet. Gynecol..

[6] Goh J.T.W., Ganyaglo G.Y.K. (2023). Sacrospinous fixation: Review of relevant anatomy and surgical technique. Int. J. Gynaecol. Obstet..

[7] Giraudet G., Ruffolo A.F., Lallemant M., Cosson M. (2023). The anatomy of the sacrospinous ligament: How to avoid complications related to the sacrospinous fixation procedure for treatment of pelvic organ prolapse. Int. Urogynecol. J..

[8] Roshanravan S.M., Wieslander C.K., Schaffer J.I., Corton M.M. (2007). Neurovascular anatomy of the sacrospinous ligament region in female cadavers: Implications in sacrospinous ligament fixation. Am. J. Obstet. Gynecol..

[9] Lee T.G., Unlu B.S. (2020). Sacrospinous ligament suspension and uterosacral ligament suspension in the treatment of apical prolapse. Gynecol. Pelvic Med..

[10] Katrikh A.Z., Ettarh R., Kahn M.A. (2017). Cadaveric Nerve and Artery Proximity to Sacrospinous Ligament Fixation Sutures Placed by a Suture-Capturing Device. Obstet. Gynecol..

[11] Enemchukwu E.A. (2019). Transvaginal suture-based repair. Urol. Clin..

[12] Meriwether K.V., Gold K.P., De Tayrac R., Cichowski S.B., Minassian V.A., Cartwright R., Rogers R.G. (2020). Joint Report on Terminology for Surgical Procedures to Treat Pelvic Organ Prolapse. Female Pelvic Med. Reconstr. Surg..

[13] Lovatsis D., Drutz H.P. (2002). Safety and efficacy of sacrospinous vault suspension. Int. Urogynecol. J. Pelvic Floor Dysfunct..

[14] Agrawal M., Kumari R., Sharma J.B., Nisha N., Manasi D., Bhatla N. (2023). Surgical Outcomes and Feasibility of Transvaginal Sacrospinous Ligament Fixation through Anterior Approach for Women with Pelvic Organ Prolapse. J. Midlife Health.

[15] Cespedes R.D. (2000). Anterior approach bilateral sacrospinous ligament fixation for vaginal vault prolapse. Urology.

[16] Zhu Q., Shu H., Du G., Dai Z. (2018). Impact of transvaginal modified sacrospinous ligament fixation with mesh for the treatment of pelvic organ prolapse-before and after studies. Int. J. Surg..

[17] Lo T.S., Ashok K. (2011). Combined anterior trans-obturator mesh and sacrospinous ligament fixation in women with severe prolapse—A case series of 30 months follow-up. Int. Urogynecol. J..

[18] Lo T.S., Kamarudin M., Chiung H.K., Rom E., Rellora L.E., Hsieh W.C. (2025). Comparison of Urinary Incontinence Occurrence Among Patients With Advanced Pelvic Organ Prolapse After Single Incision Mesh (SIM) and Anterior Mesh (A-Mesh) with Sacrospinous Ligament Fixation (SSF) Surgery at 1 Year Follow-Up Study. Low. Urin. Tract. Symptoms.

[19] Delacroix C., Allegre L., Chatziioannidou K., Gérard A., Fatton B., de Tayrac R. (2022). Anterior bilateral sacrospinous ligament fixation with concomitant anterior native tissue repair: A pilot study. Int. Urogynecol. J..

[20] Salman S., Babaoglu B., Kumbasar S., Bestel M., Ketenci Gencer F., Tuna G., Besimoglu B., Yüksel S., Uçar E. (2019). Comparison of Unilateral and Bilateral Sacrospinous Ligament Fixation Using Minimally Invasive Anchorage. Geburtshilfe Frauenheilkd..

[21] Jones C.M., Hatch K.D., Harrigill K. (2001). Unilateral and bilateral sacrospinous ligament fixation for pelvic prolapse: A nonconcurrent cohort comparison. Urogynecology.

[22] OuYang Y., Xu W., Li F., Chen Y., Yuan T., Wu X., Zhao X. (2023). Bilateral medial sacrospinous ligament suture for apical suspension through natural spaces: A single-center study with low perioperative complications. Clin. Anat..

[23] Gaultier V., Martel C., Boisramé T., Faller E., Lecointre L., Akladios C. (2023). Bilateral posterior Richter sacrospinous fixation with native tissue: Anatomical and functional results and quality of life assessment over 10 years. J. Gynecol. Obstet. Hum. Reprod..

[24] Kavvadias T., Schoenfisch B., Brucker S.Y., Reisenauer C. (2020). Anatomical and functional outcomes after hysterectomy and bilateral sacrospinous ligament fixation for stage IV uterovaginal prolapse: A prospective case series. BMC Urol..

[25] Goldberg R.P., Tomezsko J.E., Winkler H.A., Koduri S., Culligan P.J., Sand P.K. (2001). Anterior or posterior sacrospinous vaginal vault suspension: Long-term anatomic and functional evaluation. Obstet. Gynecol..

[26] Bastani P., Tayebi S., Ghabousian A., Salehi-Pourmehr H., Hajebrahimi S. (2022). Outcomes of the anterior approach versus posterior sacrospinous ligament fixation for pelvic organ prolapse. Int. Urogynecology J..

[27] Şahin F., Adan R. (2023). Comparison of anterior and posterior approach bilateral sacrospinous ligament fixation for vaginal vault prolapse. Clin. Exp. Obstet. Gynecol..

[28] Mezes C.M., Coghlan M.K., Meyer I., Russell G.B., Tranchina S.P., Daniel T.O., Snipes M., El Haraki A.S., Richter H.E., Matthews C.A. (2026). Recurrence After Anterior Versus Posterior Approach to Sacrospinous Hysteropexy. Urogynecology.

[29] Padoa A., Ziv Y., Tsviban A., Tomashev R., Smorgick N., Fligelman T. (2023). Permanent or absorbable suture material for sacrospinous ligament fixation: Does it matter?. Eur. J. Obstet. Gynecol. Reprod. Biol..

[30] Pollack B.L., Popiel P., Toaff M.C., Drugge E., Bielawski A., Sacks A., Bibi M., Friedman-Ciment R., LeBron K., Alishahian L. (2023). Permanent Compared With Absorbable Suture in Apical Prolapse Surgery: A Systematic Review and Meta-analysis. Obstet. Gynecol..

[31] ClinicalTrials.gov (2020). Loop vs. Double Suture Techniques in Sacrospinous Fixation: A Two-Year Study. https://clinicaltrials.gov/study/NCT07106866.

[32] Amiri E., Bastani P., Mallah F., Mostafaei H., Salehi-Pourmehr H. (2024). Comparison of the complications rate of different suture-passing techniques at the time of sacrospinous ligament fixation: A systematic review and meta-analysis. Arch. Gynecol. Obstet..

[33] Manning J.A., Arnold P. (2014). A review of six sacrospinous suture devices. Aust. N. Z. J. Obstet. Gynaecol..

[34] Mowat A., Wong V., Goh J., Krause H., Pelecanos A., Higgs P. (2018). A descriptive study on the efficacy and complications of the Capio (Boston Scientific) suturing device for sacrospinous ligament fixation. Aust. N. Z. J. Obstet. Gynaecol..

[35] Evangelopoulos N., Delacroix C., Abdirahman S., de Tayrac R. (2024). Safety of an anchor-based device for sacrospinous ligament fixation: A pilot case-control study. Eur. J. Obstet. Gynecol. Reprod. Biol..

[36] McKenzie C.M., Crafton C.L., Plair A., Matthews C.A. (2022). Sacrospinous Ligament Fixation Using an Anchor Versus Suture-Capturing Device: A Prospective Cohort Study. Female Pelvic Med. Reconstr. Surg..

[37] Yadav G.S., Gaddam N., Rahn D.D. (2021). A Comparison of Perioperative Outcomes, Readmission, and Reoperation for Sacrospinous Ligament Fixation, Uterosacral Ligament Suspension, and Minimally Invasive Sacrocolpopexy. Female Pelvic Med. Reconstr. Surg..

[38] Pahwa A.K., Arya L.A., Andy U.U. (2016). Management of arterial and venous hemorrhage during sacrospinous ligament fixation: Cases and review of the literature. Int. Urogynecol. J..

[39] Vodegel E.V., van Delft K.W.M., Nuboer C.H.C., Kowalik C.R., Roovers J.W.R. (2022). Surgical management of pudendal nerve entrapment after sacrospinous ligament fixation. BJOG Int. J. Obstet. Gynaecol..

[40] Yuan A.S., Propst K.A., Ferrando C.A. (2022). Postoperative pain and the need for intervention after sacrospinous ligament hysteropexy compared to colpopexy: A retrospective cohort study. Int. Urogynecol. J..

[41] Gágyor D., Pilka R., Benická A., Kališ V., Rušavý Z., Krofta L., Němec M., Mašata J. (2022). Relationship between urethrovesical junction mobility changes and postoperative progression of stress urinary incontinence following sacrospinous ligament fixation—A subanalysis of a multicentre randomized study. Ceska Gynekol..

[42] Paraiso M.F., Ballard L.A., Walters M.D., Lee J.C., Mitchinson A.R. (1996). Pelvic support defects and visceral and sexual function in women treated with sacrospinous ligament suspension and pelvic reconstruction. Am. J. Obstet. Gynecol..

[43] Vigna A., Barba M., Frigerio M. (2024). Long-Term Outcomes (10 Years) of Sacrospinous Ligament Fixation for Pelvic Organ Prolapse Repair. Healthcare.

[44] Petri E., Ashok K. (2011). Sacrospinous vaginal fixation--current status. Acta Obstet. Gynecol. Scand..

[45] Maher C., Feiner B., Baessler K., Christmann-Schmid C., Haya N., Brown J. (2016). Surgery for women with anterior compartment prolapse. Cochrane Database Syst. Rev..

[46] Yalcin Y., Demir Caltekin M., Eris Yalcin S. (2020). Quality of life and sexuality after bilateral sacrospinous fixation with vaginal hysterectomy for treatment of primary pelvic organ prolapse. Low. Urin. Tract. Symptoms.

